# Isolated Recurrent Pleural Effusions in a Patient With Rheumatoid Arthritis in Remission for Nine Years: A Case Report

**DOI:** 10.7759/cureus.35080

**Published:** 2023-02-16

**Authors:** Afra Al Dhaheri, Asma Al Neyadi, Nouf Al Naqeeb, Karthyayani P Satish

**Affiliations:** 1 Internal Medicine, Tawam Hospital, Al Ain, ARE; 2 Academic Affairs, Tawam Hospital, Al Ain, ARE; 3 Academic Affairs, Sheikh Khalifa Medical City Hospital, Abu Dhabi, ARE; 4 Medicine, Kasturba Medical College, Mangalore, IND

**Keywords:** united arab emirates, diagnosis, middle east, sulfasalazine, extraarticular manifestation

## Abstract

The development of pleural effusion in patients with active rheumatoid arthritis is a relatively common entity, yet it is uncommon in patients without clinical arthritis and other clinical features of disease flare-ups. This case report describes a 58-year-old patient with rheumatoid arthritis treated with sulfasalazine who developed recurrent large pleural effusion without clinical arthritis, after being in remission for nine years. Laboratory results showed neutrophilic leukocytosis, along with elevated inflammatory markers. Fluid analysis was suggestive of sterile exudative fluid, and adenosine deaminase of pleural fluid was negative. Culture and acid-fast bacilli of pleural fluid were both negative. Fluid cytology did not reveal any malignant cells. Chest X-ray showed right-sided pleural effusion, with underlying atelectasis. The clinical intervention included thoracentesis, piperacillin-tazobactam 4g q8 hr., prednisolone 10 mg, and sulfasalazine 1.5g. Upon hospital discharge, he was prescribed oral prednisolone 5 mg for two days, and colchicine 0.5 mg daily. After seven days, he presented with a recurrence of his symptoms and an X-ray revealed a new right-sided large pleural effusion. On the second admission, sulfasalazine was suspended, and he was switched to methotrexate. A remarkable improvement in the patient's condition was noted with an unremarkable X-ray and remained stable three months post-discharge on his following appointments as well. This report necessitates the need for the early diagnosis of a rheumatoid arthritis flare-up and the appropriate timely switch to the disease-modifying agent for better disease control.

## Introduction

Pleural involvement is a predominant thoracic complication, typically occurring in patients with known rheumatoid arthritis [1]. The classical rheumatoid pleural effusion consists of exudative fluid characterized by low pH, low glucose levels, and high lactate dehydrogenase activity [2]. Although it has been extensively reported that patients with rheumatoid arthritis can present in some cases with an extraarticular disease as the initial disease manifestation, it is rarely observed in patients without active clinical arthritis, especially subsequent recurrent large pleural effusions [3-5]. The aim of the study was to describe the effective management of isolated recurrent pleural effusions in a patient with rheumatoid arthritis in remission for nine years.

This article was previously posted to the medRxiv preprint server on December 3, 2020.

## Case presentation

A 58-year-old male, non-smoker, with a history of rheumatoid arthritis since 2011, maintained on sulfasalazine 1.5 TID, presented to the emergency department after nine years in remission (disease activity score (DAS) 2.5), with a one-week history of right-sided pleuritic chest pain. The pain was gradual, non-radiating, and aggravated by breathing. The severity was 6/10, associated with dry cough and shortness of breath. He denied any fever, rash, palpitations, night sweats, weight loss, nausea, or vomiting. He had no joint pain or body aches. There was no history of sick contacts, recent travel, or infection. The patient had long-standing seropositive rheumatoid arthritis in remission state, having developed recurrent pleural effusions after nine years of remission, with Disease Activity Score-28 for Rheumatoid Arthritis with ESR (DAS28-ESR) of 1.2 on sulfasalazine 1.5 g alone. At the time of the presentation, the patient did not have any clinical arthritis or other extraarticular manifestation with DAS28-ESR of 1.2 on sulfasalazine 1.5 g alone. He had a total of three pleural effusions over a period of one month, which were drained three times. Pleural biopsy was deferred until the patient failed methotrexate and other treatment modalities. The patient’s symptoms only resolved following stepping up his disease-modifying agent to methotrexate. Two weeks prior to presentation to this hospital’s emergency department, the patient had left leg swelling and complained of chest pain. He had visited a local private hospital twice where he was found to have right-sided pleural effusion and left leg deep vein thrombosis. The patient was investigated for hypercoagulable state and age-appropriate cancer screening such as PSA and colonoscopy. At the private hospital, he underwent pleuritic tab draining of 1L of exudative fluid and was admitted as a case of rheumatoid arthritis complicated by pleural effusion. He was prescribed therapeutic enoxaparin for five days then switched to rivaroxaban upon discharge for diagnosis of deep vein thrombosis. The patient initially improved but reported back to the private hospital after one week due to the recurrence of his symptoms. Upon the second admission, he was found to have a new right-sided pleural effusion, for which he started on oral moxifloxacin for 10 days, and oral prednisolone 5 mg for five days with minimal improvement. The patient recollected no additional investigations. His past medical history includes hypertension and dyslipidemia. He was prescribed irbesartan, hydrochlorothiazide, atorvastatin, rivaroxaban and sulfasalazine. The patient reported that for the past nine years, he was in remission (DAS28 ESR of 1.2) and did not develop any rheumatoid arthritis flare-ups or joint pain.

On physical examination, the patient was afebrile with a temperature of 37ºC and a heart rate of 77 bpm, tachypneic with a respiratory rate of 22 br/min, BP of 140/84, and O2 saturation of 97% on room air. The patient was alert, oriented, and not in acute distress. Respiratory examination revealed: stony dullness on right lower side percussion, and decreased air entry on the right side with right basal crepitation. The cardiovascular exam showed: normal S1 and S2, with no murmurs or added sounds. There was +3 pitting edema in his left leg extending to above knee level. Edema was associated with mild calf tenderness and negative Homans sign. The patient had no joint tenderness, swelling, or erythema, no rash, no mouth or nasal ulcers, and no lymphadenopathy. No clear reasons for the increase in ESR level upon discharge, and potentially contributed by the anti-rheumatic drugs that take a long time to act. The rheumatoid factor value was 66, similar to the value at the initial presentation. A subsequent lab work-up and radiological examination were ordered for the patient (Table 1).

**Table 1:** Laboratory test results for the patient ESR: erythrocyte sedimentation rate

Variable	Reference range	On admission	On Discharge
Hemoglobin (g/dl)	11.6–14.8	12.9	11.6
Hematocrit (L)	35.1–44.4	0.35	
White blood cell count (per μl)	4.5-11.0	12.8x10^9/L	9.4x10^9/L
Neutrophils (%)	0.0–2.5	83	66.7
Lymphocytes (%)	16.5–49.5	8.4	19.10
Monocytes (%)	2.0–10.0	8.2	11.10
Eosinophil (%)	0.0–8.5	0.20	2.70
Platelet count (per μl)	158,000–348,000	256,000	255,000
Sodium (mmol/L)	135–145	138	137
Potassium (mmol/L)	3.6–4.8	3.6	4.3
Chloride (mmol/L)	101–108	103	100
Creatinine (micromol/L)	61-106	113	102
Urea nitrogen (mmol/L)	2.80-8.10	4.9	4.30
C‐reactive protein (mg/dl)	Normal high <=5	224	90
ESR		64	98

**Figure 1:**
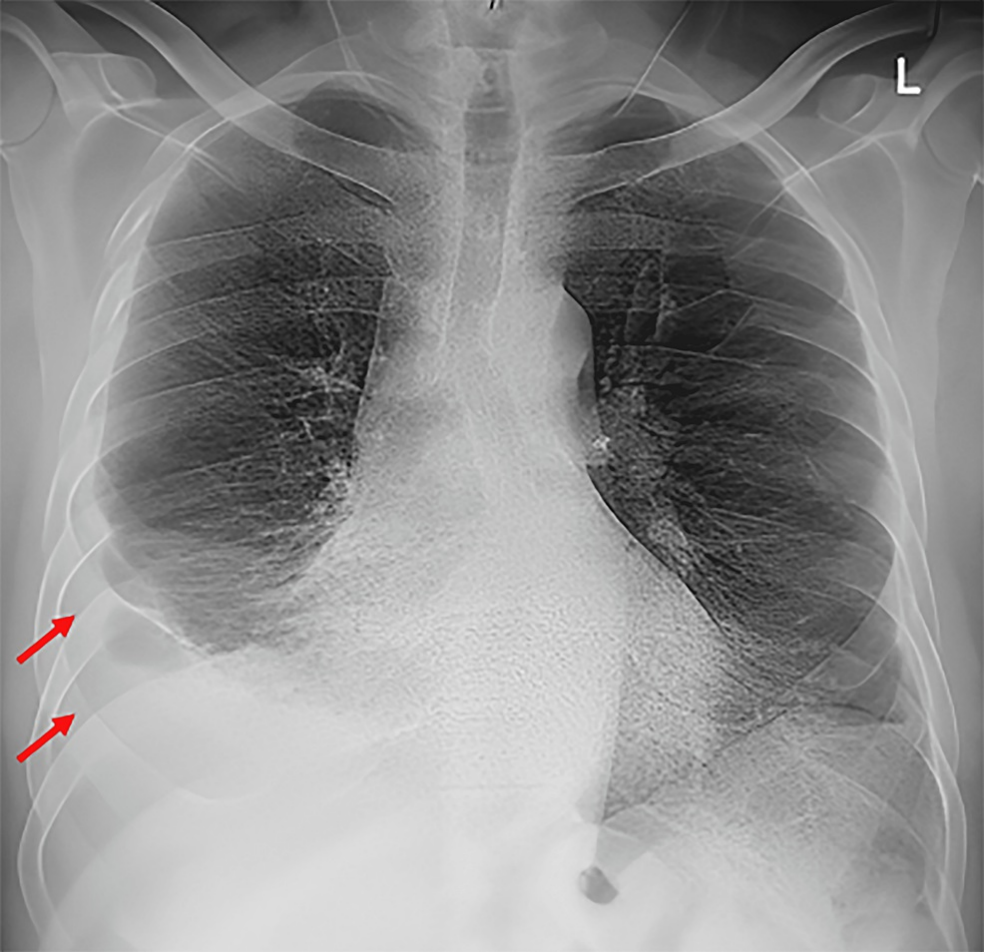
Chest radiogram showing a right-sided pleural effusion with underlying atelectasis

**Figure 2:**
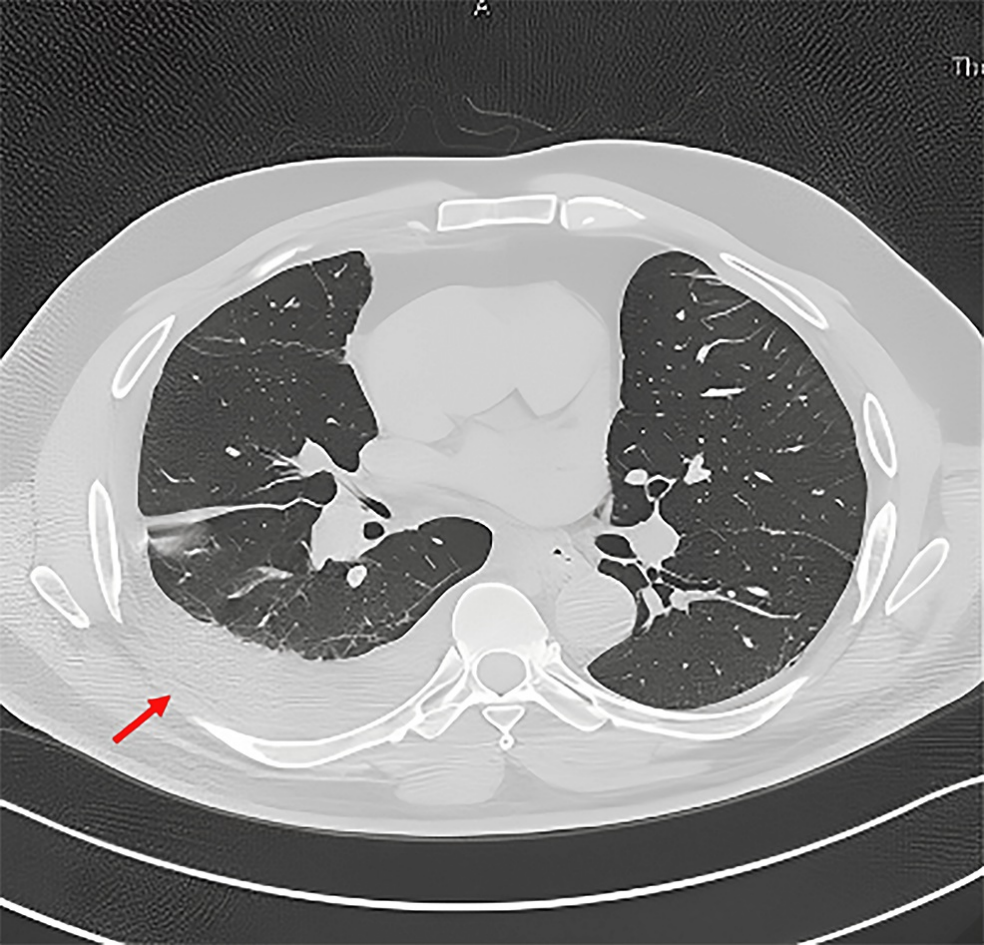
CT thorax showing a mild right-sided pleural effusion with lower lung segmental collapse

The chest X-ray showed right-sided pleural effusion, with underlying atelectasis (Figure 1). CT thorax w/o contrast post pleural tap (Figure 2) showed bilateral basal atelectasis, fibrotic bands, minimal right pleural effusion, and a trace of pleural effusion on the left side. There was no evidence of chronic lung disease. Pan CT to rule out malignancy returned negative and the CT pulmonary embolus (CTPE) was negative.

Laboratory results showed neutrophilic leukocytosis, along with elevated inflammatory markers with an ESR of 64. ECG and troponin were done to rule out cardiac causes of chest pain, which were within normal (Table 1) Fluid analysis was suggestive of sterile exudative fluid. Adenosine deaminase of pleural fluid was negative. Culture and acid-fast bacilli of pleural fluid were both negative. Fluid cytology did not reveal any malignant cells (Table 2).

**Table 2:** Pleural fluid assessment LDH: lactate dehydrogenase

Fluid analysis (Pleural fluid)	Finding
Appearance	Turbid
Color	Yellow
RBC	4,000 cell/mm3
WBC	11,335
Segs	78
Lymphocyte	14
Monocytes	6
Eosinophil	2
Albumin	23g/L
Protein	48 g/L
Glucose	1.7 mmol/L
Urea	5.4 mmol/L
LDH	692 IU/L
Uric acid	356

The patient was initially managed with thoracentesis, Piperacillin-tazobactam 4g q8 hr, prednisolone 10 mg, and sulfasalazine 1.5 g as a case of rheumatoid arthritis (RA) complicated by pleural effusion with possible underlining pneumonia (the patient had no history of cough or purulent sputum). After this treatment plan, he showed some improvement and was discharged with oral prednisolone 5 mg for two days and colchicine 0.5 mg daily, as a treatment for pleurisy, as advised by the primary internal medicine team. Seven days after discharge, he presented to the emergency department with a recurrence of his symptoms. A repeat X-ray revealed a new right-sided large pleural effusion. He was tapped again, and analysis showed exudative pleural effusion. Upon his second admission, sulfasalazine was suspended, and he was switched to methotrexate 15 mg once weekly and folic acid 5 mg once weekly, to achieve better control of his disease. The patient remarkably improved after starting methotrexate and cessation of sulfasalazine. He was seen in the rheumatology clinic two weeks later, on methotrexate 15 mg and folic acid 5 mg weekly only. He was feeling well, improved clinically, and his follow-up X-rays showed no recurrence. His ESR level during the outpatient rheumatology clinic visit was 98 (there were no clear reasons for the increase in the ESR, although methotrexate takes some time to curb inflammation). The patient remained stable three months post-discharge on his following appointments as well.

## Discussion

It is estimated that the incidence of pleural effusion in rheumatoid arthritis patients ranges between 3% and 5% [2]. The main causes of pleural effusion in such patients include chronic inflammation, local immune complex formation, an active infection due to immunosuppressive agents, and drug-induced pleural disease [3]. Based on prior studies, older patients, men, patients with higher levels of rheumatoid factor (RF), and late-onset disease are at higher risk of developing pleural effusions [2]. Upon presentation, patients usually are asymptomatic with small to medium unilateral pleural effusion that resolves spontaneously without the need for any treatment. It is also noted that pleural effusion can precede joint disease in rheumatoid arthritis, however, an extraarticular manifestation of rheumatoid arthritis rarely presents in patients with the established disease who do not have an acute flare-up or clinical arthritis [4].

The present case is interesting and unique, given the fact that the patient presented after nine years of remission (DAS 2.51), with recurrent symptomatic large pleural effusions. During the past years, he had minimal articular symptoms and no clinical arthritis at the time of presentation. Upon investigation, his imaging studies were normal aside from bilateral effusions larger on the right side. In addition, infectious and malignant etiologies were ruled out by comprehensive imaging and labs. Typically, rheumatoid arthritis patients with pleural effusion improve spontaneously without any treatment or with one-time thoracentesis with or without systemic corticosteroids [2]. In our case, the patient was initially managed with pleural tapping and a course of oral steroids for one week to reduce ongoing inflammation, along with a course of antibiotics for possible underlining infection. However, the patient experienced recollection of right pleural effusion despite two thoracocentesis procedures. The thoracic surgeon was consulted for pleural biopsy and pleurodesis, the thoracic team advised waiting if adding immunosuppressive treatment failed to consider it, but the patient didn't show signs of recurrence of pleural effusion after starting him on methotrexate. The subsequent change of the disease-modifying agent from sulfasalazine to methotrexate 15 mg enabled better control of the patient’s rheumatoid arthritis. The introduction of methotrexate allowed control of disease flare-ups, and the patient did not develop any subsequent effusions.

It is assumed that the patient experienced sulfasalazine-induced lung toxicity (5). It has been reported that patients on sulfasalazine with an average dose of 3 g and an average duration of 17.8 months presented with pulmonary disease [5]. Additionally, it was noted that following imaging, 92% have pulmonary infiltrates and one with minimal effusion (5). The CT scan for five of the reported cases showed ground-glass opacities. All patients who were withdrawn from the drug showed improvement and resolution of symptoms with an average of slightly over six weeks. The exact reason for our patient’s improvement following methotrexate is unknown, neither is it clear whether sulfasalazine toxicity had a role to play, especially given the fact that the CT findings could not be corroborated with any previous observations.

## Conclusions

It is to be noted that acute recurrent pleural effusions in patients with rheumatoid arthritis can be a rare presentation of acute disease flare-up despite the absence of clinical arthritis. This is especially relevant in the absence of secondary causes such as infection, malignancy, and drug toxicity. This report necessitates the need for early diagnosis, and a rapid switch to the optimal disease-modifying agent, to obtain better disease control and limit a variety of associated complications. This is perhaps the first case reported on pleural effusion in rheumatoid arthritis patients in long remission from the Middle Eastern region.
